# Specialized mental healthcare use for common mental disorders and prescription of antidepressants before and during the COVID-19 pandemic among working-age refugees and Swedish-born individuals – a nationwide register-based study

**DOI:** 10.1186/s12889-025-22028-4

**Published:** 2025-03-03

**Authors:** Vera Atarodi, Ellenor Mittendorfer-Rutz, Daniel Morillo-Cuadrado, Roberto Mediavilla, Mireia Felez-Nobrega, Anna Monistrol-Mula, Pierre Smith, Vincent Lorant, Papoula Petri-Romão, Marit Sijbrandij, Anke B. Witteveen, Irene Pinucci, Matteo Monzio Compagnoni, Claudia Conflitti, Giulia Caggiu, Maria Melchior, Cécile Vuillermoz, Jakob Bergström, Katalin Gémes

**Affiliations:** 1 Department of Clinical Neuroscience, Karolinska Institutet, Stockholm, Sweden; 2 School of Psychology, Universidad Nacional de Educación a Distancia (UNED), Madrid, Spain; 3 Instituto de Investigación Sanitaria del Hospital Universitario La Princesa (IIS-Princesa), Madrid, Spain; 4 Centro de Investigación Biomédica en Red de Salud Mental (CIBERSAM), Instituto de Salud Carlos III, Madrid, Spain; 5 Department of Psychiatry, Universidad Autónoma de Madrid, Madrid (UAM), Spain; 6 Group of Epidemiology of Mental Disorders and Ageing, Institut de Recerca Sant Joan de Deu (IRSJD), Parc Sanitari Sant Joan de Deu, Sant Boi de Llobregat, Spain; 7 Department of Epidemiology and Public Health, Sciensano Belgian Institute of Health, Brussels, Belgium; 8 Institute of Health and Society, Université Catholique de Louvain, Brussels, Belgium; 9 Leibniz Institute for Resilience Research, Wallstr 7 55122, Mainz, Germany; 10 Department of Clinical, Neuro- and Developmental Psychology, Amsterdam Public Health Institute and World Health Organization Collaborating Center for Research and Dissemination of Psychological Interventions, Vrije Universiteit, Amsterdam, Netherlands; 11 Department of Human Neurosciences, Sapienza University of Rome, Rome, Italy; 12 Department of Statistics and Quantitative Methods, University of Milano-Bicocca, Milan, Italy; 13 Department of Mental Health and Addiction Services, ASST Lecco, Lecco, Italy; 14 Equipe de Recherche en Epidémiologie Sociale (ERES), Institut Pierre Louis d’Epidémiologie Et de Santé Publique (IPLESP), INSERM, Sorbonne Université, Faculté de Médecine St Antoine, Paris, France

**Keywords:** Common mental disorders, COVID-19, Interrupted time-series, Population-based cohort, Mental health, Refugees

## Abstract

**Background:**

It is known that refugees have an elevated risk of common mental disorders (CMDs, including depression, anxiety, and stress-related disorders). The effect of the coronavirus disease pandemic on healthcare use due to CMDs in refugees is yet unknown, especially in socioeconomically deprived groups. We conducted a population-wide study comparing specialized healthcare use for CMDs and antidepressant prescriptions before and during the pandemic in refugees and Swedish-born, and investigated differences by labor market marginalization and education.

**Methods:**

An interrupted time series analysis of quarterly cohorts (2018.01.01–2021.12.31) of all refugees and Swedish-born, aged 19 to 65 was applied. Information on outcome measures and covariates were linked individually from administrative registers. We applied interrupted time series and estimated incidence rate ratios (IRR) of the incidence rates (IR) and their corresponding confidence intervals (CI) before and during the pandemic.

**Results:**

A total of 4,932,916 individuals, of whom 488,299 (9.9%) were refugees, were included at baseline. We observed a 3% (95% CI: 1%, 5%) quarterly increase in trends of healthcare use due to CMDs in refugees, but no changes in Swedish-born individuals. The IRRs were larger in refugees whose labor market position was marginalized (IRR: 6%, (3%, 9%)), and refugees with low education level (IRR: 4% (1%, 7%)). There were no substantial changes in antidepressant prescription.

**Conclusion:**

Refugees, especially those already in a marginalized position, had increased CMD-related mental healthcare use during the pandemic. Strategies to meet the mental health care needs of marginalized refugees are of outmost public health importance.

**Supplementary Information:**

The online version contains supplementary material available at 10.1186/s12889-025-22028-4.

## Background

The coronavirus disease 2019 (COVID-19) pandemic is one of the most significant recent public health emergencies leading to a strong increase in mortality and adverse somatic health outcomes around the world [1]. Fear of infection, public health mitigation strategies to decrease interpersonal contacts [2, 3] and the disruption of mental health and social care services [4, 5] are all factors that might have increased the risk of mental ill-health. However, so far current evidence on the COVID-19 pandemic’s effect on mental health is contradictory. Population-based studies find no change or even a decrease in the rate of severe mental disorders and suicide in several countries [6–9]. Recent meta-analyses of longitudinal European studies concluded an evidence of high-moderate certainty that the prevalence of depression, generalized anxiety disorder and non-specific mental health problems increased slightly during the first months of the pandemic, but remained stable or decreased to pre-pandemic levels later during the pandemic [6, 10]. Studies investigating patterns in healthcare use in various European countries have either found no changes or even a reduction in healthcare use due to depression, anxiety and stress-related disorders (i.e. common mental disorders, CMDs) at the beginning of the pandemic [6, 11, 12]. These trends have been followed by a later slight increase, and for some services this increase was above the pre-pandemic level [11, 13].

While this overall picture does not support a major increase in the rate of healthcare use for CMDs during the pandemic in the general working age population, concerns are emerging that some vulnerable marginalized groups, such as refugees, might have been disproportionately affected [9, 14, 15]. Various pre- and postmigration factors, such as traumatic experiences, lower socioeconomic status, lower labor market attachment, cultural and language barriers, poorer health literacy and likely discrimination are refugee-specific risk factors that may contribute to a higher vulnerability to mental ill-health [16, 17].

Despite having a higher prevalence of CMDs compared to the host population, refugees underutilize mental health care services [18] due to a number of barriers such as language problems, stigma related to mental disorders and restrictions to health care in the host country [19]. Lack of knowledge of the health care system in the host country and differences in the experience and expression of mental disorders in the country of origin additionally contribute to lower levels of health care seeking of refugees with mental health complaints. It is likely that use of mental health care was additionally adversely affected in refugees by the changes in services provision during the pandemic, which occurred despite efforts in compensating pandemic-related disruptions in services by providing telemedicine services [4, 5, 20]. It is, however, also possible that the mental health care use during the pandemic increased in refugees. One reason for such an increase might be the pandemic’s strong effect on labor market marginalization, which is more prevalent among refugees than in the host population. Due to the known link between labor market marginalization and poor mental health, refugees’ mental health might have been unevenly adversely affected and, in turn, might have resulted in increased mental health care use [4, 21–23]. Furthermore, this negative effect on mental health might be even more pronounced among refugees who were in a more socially deprived position at the beginning of the pandemic. For instance, refugees already marginalized in the labor market, such as on long-term unemployment or on disability pension or having low level of education, as these factors are associated with poor mental health and are more prevalent in refugees compared with Swedish-born [15, 22].

It is, therefore, crucial to study patterns of mental health care use in refugees during the pandemic. Still, until recently the effect of the COVID-19 pandemic on refugees’ mental health is rather unexplored [9, 24–26] and we could not find any population-wide studies on changes in health care use due to CMDs, before and during the pandemic, in refugees. The use of nationwide registers with information on mental health care use due to CMDs minimalizes selection bias and allows to draw inference to the whole population of refugees and those born in Sweden. Furthermore, as antidepressant medication is prescribed in primary health care, using information on those in addition to specialized healthcare use due to CMDs allows to cover information on less severe cases. Hereby, by using administrative national registries the whole spectrum of care for patients with CMDs can be covered.

The Swedish mitigation strategy differed initially from other countries, e.g., there was no lockdown, kindergarten and primary schools stayed open, and the public health strategy was primarily focused on voluntary measures and the individual’s responsibility [27]. There were, however, some measures taken, as banning visits at elderly care residents, limiting public gatherings, recommending online classes for those older than 17 years and remote working [27]. However, similarly to other countries the healthcare system was impacted both by the increased number of severe COVID-19 cases, and with limiting access to normal care to prevent spreading of the virus. To provide safe care there were several changes introduced, such as the transition to telemedicine in primary health, which was relative fast given the already higher level of digitalization [28]. Furthermore, Sweden had a focus from the beginning of the pandemic to mitigate the possible mental health impact of the pandemic by advising good public mental health practice even with some pandemic-related limitations and advising care seeking behavior due to mental health problem in the general population. This particular setting allows to study the impact of a particular public health strategy on mental health in the general and also in more vulnerable populations, such as refugees, and those who with an already low attachment to the labor market, and it will contribute to the growing body of literature concerning the consequences of the pandemic. To address the described current knowledge gaps, we conducted a Swedish nationwide register-based study to compare incidence trends in specialized health care use due to CMDs and in antidepressant prescription before and during the pandemic in refugees and Swedish-born. We also investigated whether being already marginalized in the labor market or having low education modify the studied associations in refugees and Swedish-born individuals. We hypothesize that individuals with a low attachment to the labor market are more socially marginalized and therefore at greater risk of decreasing mental health as they might be more affected by the negative consequences of limited access to health and social care due to language and cultural barriers and, increased social isolation.

## Methods

### Study populations

The observation period, from January 1, 2018, to December 31, 2021, was divided in quarters. Thus, the follow-up period consisted of sixteen quarters, and at the beginning of each considered quarter, a cohort of patients who met the inclusion criteria, described below, was identified and followed till the end of the quarter. All individuals aged 19–64 years, registered and living in Sweden at the start of a quarter and at least one year prior to the quarter of cohort entry were included. Individuals included had to be either registered as born in Sweden or as born in a country outside of Sweden with ‘refugee’ as reason for residency in Sweden.

This is a register linkage study. Information on outcomes and covariates was obtained from 5 administrative registers: (i) the Longitudinal database for integration studies, STATIV, provided by Statistics Sweden, was used to obtain information on reason for residence [29]. (ii) The Longitudinal integrated database for health insurance and labor market studies (LISA), held by Statistics Sweden, contains information on all individuals who are residing in Sweden on a yearly basis. It provided information on age, sex, country of birth, educational level, living area and yearly number of days on unemployment benefits [30]. (iii) The Micro Data for Analysis of the Social Insurance database (MIDAS), held by the Social Insurance Agency, was used for information on net days of sickness absence and disability pension [31]. (iv) The date and diagnoses of in- and specialized outpatient care due to CMDs were obtained from the National Patient Register (NPR) [32], and (v) antidepressants dispensation was provided in the Prescription Drug Register. The latter registers are both held by the National Board of Health and Welfare [33]. All these registers were linked through a unique de-identified Swedish personal identification number.

### Variables

#### Outcomes

The outcomes were defined as “incident” events. Events within one year prior to the start of the observed quarter were therefore not counted. *Use of specialized mental health care due to common mental disorders* was defined as an incident of inpatient or specialized outpatient care event according to the following International Classification of Diseases 10th version (ICD-10) [34] codes: F32-F34 (depressive disorder), F43 (stress related disorder), F40-F42, F44, F48 (neurotic disorders). *Antidepressants prescription* was defined as an incident of prescription and dispensation by the N06A Anatomic Therapeutic Chemicals (ATC) code [35]. The outcomes were assessed quarterly.

#### Covariates

*Labor market marginalization,* a concept used in studies to capture a social insurance perspective [36]*,* was defined as more than 365 days of unemployment, sickness absence or disability pension (net days), in the year prior to the observed quarter. For employees, only the sickness absence days from day 15 are included as the employer pays the first 14 days and the first day is a qualifying day. The cut-off of 365 days was chosen in order to capture permanent labor market marginalization that was not caused by the temporary effect of the pandemic on the labor market (Supplementary material). *Education* assessed at baseline was categorized as less than or equal to nine, or more than nine years of completed education, as that is the level of compulsory schooling in Sweden. Missing values in education are more common in refugees and often indicate none or elementary schooling and therefore categorized as less than nine years of education. All other variables were used for descriptive statistics only and coded as indicated in Table 1.

**Table 1:** Descriptive statistics of sociodemographic characteristics in the refugee and Swedish-born population, at baseline

**Characteristics**	**Category**	**Refugees** *N* = 488,299		**Swedish-born** *N* = 4,444,617	
		N	%	N	%
Sex	Women	216 187	44.3	2 170 597	48.8
	Men	272 112	55.7	2 274 020	51.2
Age-groups (years)	19–25	77 736	15.9	646 218	14.5
	26–35	136 836	28.0	1 008 618	22.7
	36–45	108 143	22.1	904 394	20.3
	46–55	97 569	20.0	1 048 612	23.6
	56–64	68 015	13.9	836 775	18.8
Years of education	≤ 9	165 962	34.0	511 945	11.5
	> 9	322 337	66.0	3 932 672	88.5
Level of urbanization	Cities	243 766	49.9	1 699 065	38.2
	Middle size towns and villages	178 552	36.6	1 862 555	41.9
	Rural areas	65 981	13.5	882 997	19.9
Sickness absence	Yes	38 499	7.9	490 192	11.0
	No	449 800	92.1	3 954 452	89.0
Disability pension	Yes	25 363	5.2	234 075	5.3
	No	462 936	94.8	4 210 542	94.7
Unemployment	Yes	156 502	32.1	276 563	6.2
	No	331 797	67.9	4 168 054	93.8
Labor market marginalization^a^	Yes	33 617	6.9	203 945	4.6
	No	454 682	93.1	4 240 672	95.4

Baseline: 2017, i.e. before the first quarter of 2018. Statistics presented in counts and percentages

^a^Labor market marginalization was defined as > 365 net days of sickness absence, disability pension or unemployment one year prior the outcome measure

### Statistical analysis

We conducted an interrupted time series (ITS) analysis to compare time trends before and during the COVID-19 pandemic. The World Health Organization announced the pandemic in March 11, 2020 [1] hence, we defined the interruption (the outbreak of the pandemic) as the time point between the end of the first quarter (January 1, 2020—March 30) and the beginning of the second quarter (April 1, 2020—June 30) of 2020.

Data was aggregated for each quarter for incidences of both outcomes and total follow-up time. We then evaluated the changes of incidence rates (IRs) by conducting a log-linear Poisson regression using general linear models to compare the rate of specialized mental health care use due to CMDs and antidepressant prescription before and during the pandemic. All models included two time-variables, one estimating the linear change in IRs before the interruption and one estimating the difference in linear change in IRs after and before the interruption. IRs and incidence rate ratios (IRRs) were presented with 95 percent confidence intervals (CIs). The model also included an offset variable, which was the log of the follow up time in each quarter, taking into account different follow-up times due to death or migration.

As the outcomes had a strong seasonal variability, we adjusted for it by incorporating the mean of the outcomes in each quarter in the model as a covariate. Due to dependency of the data, as individuals could be included in more than one quarter, a Sandwich estimator was used to adjust the standard errors estimated by the general linear models. The predicted quarterly IRs adjusted by seasonal variation were calculated by using marginal effects at the mean. We also conducted analyses stratified by labor market marginalization and by baseline level of education both within refugees and Swedish-born. As the mitigation measures might affect the number of newcomers in the country, we conducted a sensitivity analysis by the “length of stay in Sweden”, in order to distinguish between refugees who have been staying longer in Sweden than compared with shorter or equal to 5 years. Data management was conducted in Stata version 17 and statistical analysis were conducted in R version 4.2.2.

## Results

### Descriptive statistics

There was a total of 4,932,916 individuals, 488,299 of whom refugees, included in this study in 2017 (Table 1). Most of the refugees were born in Asia (58.2%), Europe (19.6%) and Africa (18.9%). Low education was more prevalent among refugees (34%) than Swedish-born (13.7%). There were 4.2% missing values on education among refugees and 0.6% among the Swedish-born. Overall, 11.0% of Swedish-born compared with 7.9% of refugees had any sickness absence days, while 32.1% of refugees compared with 6.2% of Swedish-born had at least one registered unemployment day in 2017.

### Specialized mental health care use for CMDs

**Fig. 1:**
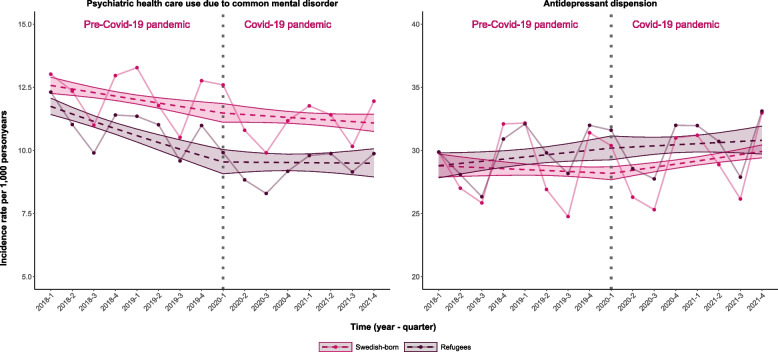
Incidence rates and trends of outcomes studied before and during the COVID-19 pandemic. Note. Observed incidence rates and trends in incidence rates of specialized mental health care use for common mental disorders and prescription of antidepressants before and during the COVID-19 pandemic for refugees and Swedish born. The dots represent observed values; dashed lines represent linear regression predicted values and the strict lines represent 95% confidence intervals of the predicted values. The vertical dashed line illustrates the interruption, i.e. start of the COVID-19 pandemic

Comparing the IR trends for specialized mental health care use for CMDs in refugees before and during the pandemic revealed a 2.5% quarterly increase (95% CI: 0.7%, 4.5%) during the pandemic (Fig. 1, Table 2). The IR trend before the pandemic was -2.6% (95% CI: -3.3, -1.8) while the IR trend during the pandemic was -0.1 (-1.3;1.1). Whereas, in Swedish-born the comparison between the IR trends before and during the pandemic was 0.6% (-0.6%, 1.9%). The IR trend among Swedish-born was -1.1 (-1.7, -0.5) before the pandemic and -0.5 (-1.2;0.2) during the pandemic. For observed IR of specialized mental health care use for CMDs see Additional Table 1 in Additional files 1.

**Table 2:** Quarterly changes of incidence rates and incidence rate ratios for outcomes observed

	Quarterly changes of IRs (%) before the pandemic	Quarterly changes of IRs (%) during the pandemic	IRRs comparing quarterly changes of IRs (%) during vs before the pandemic
	**Trend IR (95% CIs)**	**Trend IR (95% CIs)**	**Trend IRR (95% CIs)**
**Specialized health care use for common mental disorders**			
Refugees	-2.6 (-3.3;-1.8)	-0.1 (-1.3;1.1)	2.5 (0.7;4.5)
Swedish-born	-1.1 (-1.7;-0.5)	-0.5 (-1.2;0.2)	0.6 (-0.6;1.9)
**Antidepressant prescription**			
Refugees	0.3 (-0.2;0.9)	0.3 (-0.4;1.0)	0.0 (-1.1;1.0)
Swedish-born	-0.3 (-0.8;0.2)	0.8 (0.4;1.2)	1.1 (0.3;1.9)

Quarterly changes of incidence rates (IRs) in %, incidence rate ratios (IRRs) in % with 95% confidence intervals (CIs) for specialized psychiatric health care use for common mental disorder and antidepressant prescription comparing trends before and during the pandemic in the refugees and Swedish-born

**Fig. 2:**
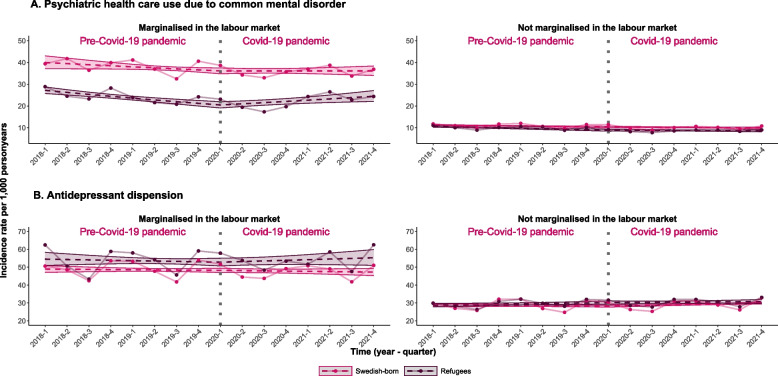
Incidence rates and trends of outcomes studied stratified by labor market marginalization. Note. Observed incidence rates and trends in incidence rates of specialized mental health care use for common mental disorders and prescription of antidepressants before and during the COVID-19 pandemic for refugees and Swedish born stratified by labor market marginalization. The dots represent observed values; dashed lines represent linear regression predicted values and the strict lines represent 95% confidence intervals of the predicted values. The vertical dashed line illustrates the interruption i.e. start of the COVID-19 pandemic

**Fig. 3:**
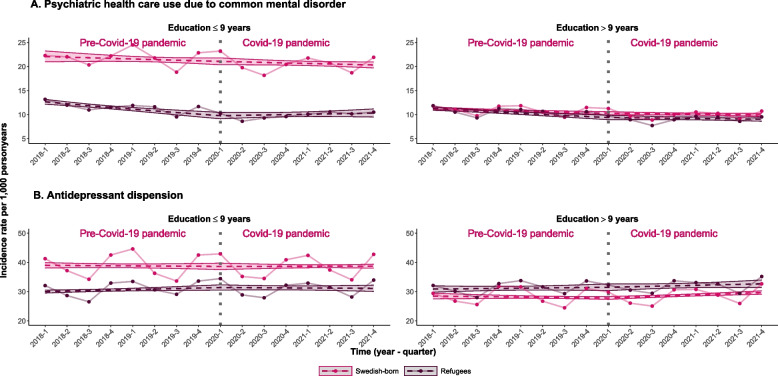
Incidence rates and trends of outcomes studied before and during the pandemic stratified by education. Note. Observed incidence rates and trends in incidence rates of specialized mental health care use for common mental disorders and prescription of antidepressant before and during the COVID-19 pandemic for refugees and Swedish born stratified by education. The dots represent observed values; dashed lines represent linear regression predicted values and the strict lines represent 95% confidence intervals of the predicted values. The vertical dashed line illustrates the interruption i.e. start of the COVID-19 pandemic

When we stratified our analyses by labor market marginalization, we found that among marginalized refugees the quarterly IR trend was -4% (95% CI: -5%, -3%) before the pandemic, which changed to 3% (95% CI: 1%, 4%) during the pandemic, resulting in a 6.3% relative change of quarterly IR trends (95% CI: 3.2%, 9.5%) (Fig. 2, Table 3). The relative changes in quarterly IR trends before and during the pandemic were 1.8% (95% CI: -0.2%, 3.7%) in refugees who were not marginalized on the labor market and 0.5% (95% CI: -0.7;1.6) in Swedish-born. Stratifying the analyses by level of education showed similar results. In refugees with a low level of education we observed a -3.2% (95% CI: -4.3%, -2.0%) quarterly IR trend in specialized mental health care use for CMDs before the pandemic, which increased to 0.8% (95% CI: -0.8%, 2.4%) during the pandemic, resulting in a 4.1% relative change in quarterly IR trends (95% CI: 1.4%, 6.8%) (Fig. 3, Table 4).

**Table 3:** Quarterly changes of incidence rates and incidence rate ratios for outcomes observed stratified by labor market marginalization

	**Labor market marginalized**^a^			**On the labor market**		
	Quarterly changes of IRs (%) before the pandemic	Quarterly changes of IRs (%) during the COVID-19 pandemic	IRRs comparing quarterly changes of IRs (%) during vs before the pandemic	Quarterly changes of IRs (%) before the pandemic	Quarterly changes of IRs (%) during the pandemic	IRRs comparing quarterly changes of IRs (%) during vs before the pandemic
	Trend IR (95% CIs)	Trend IR (95% CIs)	Trend IRR (95% CIs)	Trend IR (95% CIs)	Trend IR (95% CIs)	Trend IRR (95% CIs)
**Specialized psychiatric health care use for common mental disorders**						
Refugees	-3.5 (-5.0;-2.0)	2.6 (0.8;4.4)	6.3 (3.2;9.5)	-2.1 (-3.0;-1.3)	-0.4 (-1.7;0.8)	1.8 (-0.2;3.7)
Swedish-born	-1.3 (-2.3;-0.3)	0.0 (-1.1;1.0)	1.3 (-0.6;3.2)	-1.0 (-1.6;-0.4)	-0.6 (-1.2;0.1)	0.5 (-0.7;1.6)
**Antidepressant prescription**						
Refugees	-0.4 (-1.6;0.9)	0.7 (-0.4;1.8)	1.1 (-0.9;3.1)	0.6 (-0.1;1.2)	0.3 (-0.5;1.1)	-0.3 (-1.5;1.0)
Swedish-born	-0.2 (-0.8;0.5)	-0.2 (-0.9;0.4)	-0.1 (-1.2;1.1)	-0.3 (-0.8;0.2)	0.9 (0.5;1.3)	1.1 (0.3;1.9)

Quarterly changes of incidence rates (IRs) in %s before and during the COVID-19 pandemic, and incidence rate ratios (IRRs) in %s with 95% confidence intervals (CIs) for specialized psychiatric health care use for common mental disorder and antidepressant prescription comparing before and during the pandemic trends in the refugees and Swedish-born stratified by labor market marginalization

^a^Labor market marginalization was defined as > 365 net days of sickness absence, disability pension or unemployment one year prior the outcome measure

**Table 4:** Quarterly changes of incidence rates and incidence rate ratios for outcomes observed stratified by education

	**Less than 10 years of completed education**			**10 years or more of completed education**		
	Quarterly changes of IRs (%) before the pandemic	Quarterly changes of IRs (%) during the pandemic	IRRs comparing quarterly changes of IRs (%) during vs before the pandemic	Quarterly changes of IRs (%) before the pandemic	Quarterly changes of IRs (%) during the pandemic	IRRs comparing quarterly changes of IRs (%) during vs before the pandemic
	Trend IR (95% CIs)	Trend IR (95% CIs)	Trend IRR (95% CIs)	Trend IR (95% CIs)	Trend IR (95% CIs)	Trend IRR (95% CIs)
**Specialized psychiatric health care use for common mental disorders**						
Refugees	-3.2 (-4.3;-2.0)	0.8 (-0.8;2.4)	4.1 (1.4;6.8)	-2.2 (-3.0; -1.5)	-0.5 (-1.6;0.5)	1.7 (0.0;3.5)
Swedish-born	-0.6 (-1.4;0.3)	-0.5 (-1.3;0.4)	0.1 (-1.5;1.7)	-1.2 (-1.8; -0.6)	-0.4 (-1.1;0.2)	0.8 (-0.4;1.9)
**Antidepressant prescription**						
Refugees	0.6 (0.2;1.0)	0.2 (-0.4;0.9)	-0.6 (-1.5;0.3)	0.2 (-0.4;0.9)	0.5 (-0.3;1.3)	1.2 (0.4;2.0)
Swedish-born	-0.1 (-0.6;0.4)	-0.3 (-0.8;0.2)	0.2 (-0.7;1.1)	-0.3 (-0.8;0.2)	0.9 (0.5;1.3)	0.3 (-1.0;1.6)

Quarterly changes of incidence rates (IRs) in %s before and during the COVID-19 pandemic, and incidence rate ratios (IRRs) in %s with 95% confidence intervals (CIs) for specialized psychiatric health care use for common mental disorder and antidepressant prescription comparing before and during the pandemic trends in the refugees and Swedish-born stratified by level of education

### Antidepressant prescription

The modelled IR trends for antidepressant prescription were 0.3% both before (95% CI: -0.4;1.0) and during (95% CI: -0.2; 0.9) the pandemic among refugees (Fig. 1 and Table 2). For the Swedish-born the modelled IR trends for antidepressant prescription were -0.3% (95% CI: 0.8;0.21%) before the pandemic and 0.8% (95% CI: 0.4%, 1.2%) during the pandemic. The IRR showed a relative 1.1% (95% CI: 0.3%, 1.9%) quarterly IR increase during the pandemic among the Swedish-born, compared to the IR trend before and 0.0% (95% CI: -1.1%, 1.0%) for refugees (Fig. 1 and Table 2).

Stratifying by labor market marginalization showed similar results as in the main analyses, revealing minimal changes in quarterly IR trends of antidepressant prescription before and during the pandemic in both refugees and Swedish born and in both strata (Fig. 2, Table 3). After stratification by level of education, the results were similar and the IRRs comparing quarterly IR trends before and during the pandemic were 0%-1% in all groups (Fig. 3, Table 4).

The sensitivity analysis, stratifying the refugee population on length of stay in Sweden, showed that there was a 2.6% (95% CI: 0.6, 4.7%) change in trends of specialized psychiatric health care for CMD among refugees who had stayed in Sweden longer than five years and 1.6% (95% CI: -0.6, 3.8%) among refugees who had stayed in Sweden less than or equal to five years. Concerning antidepressant prescription, the sensitivity analysis found that in the group of refugees by length of stay, where there was a -1.9% (95% CI: -3.4, 0-0.7%) change in trends for the group of refugees who had been in Sweden five years or less but no change in those who had been in Sweden longer than five years 0.1% (95% CI: -0.7, -1%). For results from sensitivity analysis see Additional Table 3 and Additional Fig. 1 in Additional files 1.

## Discussion

### Summary of results

After the outbreak of the COVID-19 pandemic, there was a relative increase in specialized mental health care use for CMDs among refugees, but no related change among Swedish-born. The increasing trend in specialized mental health care use for CMDs was most pronounced among refugees who had a low attachment on the labor market, or had a low educational level. Concerning antidepressants prescription, there were no substantial changes in the trends before and during the COVID-19 pandemic neither in the refugee nor in the Swedish-born population.

### Comparison with previous findings

In line with our results among Swedish-born people, previous studies mostly covering the beginning of the pandemic found no change or a slight decrease of the use of mental health care services during the pandemic [6, 11, 12]. A recent study including Swedish data found a drop in primary health care use due to CMDs at the beginning of the pandemic, which stayed low in Sweden during the entire 2020. In Norway and the Netherlands, primary health care use decreased at the beginning of the pandemic but increased to pre-pandemic levels later, while in Latvia it increased over the pre-pandemic level [11]. Another study from Norway reported no overall increase of primary health care use for any mental disorder during the first year of the pandemic [12]. Moreover, register-based studies from the UK, found no evidence of increasing trends in incident health care use for depression, anxiety and post-traumatic stress disorder in the first year of the pandemic [6]. Finally, a study conducted in Denmark investigating admission trends in inpatient psychiatric care for severe mental disorders up to March 2021, reported lower levels of admission rates during the observed period than pre-pandemic levels [37]. Compared to these studies, we had the possibility to follow the trends in mental health care use over a longer time period, i.e. up to the end of 2021. It is possible that mental health care needs of the Swedish-born population increased during the beginning of the pandemic and disrupted services could initially not meet these needs. Still, given the long follow-up time in our study, it is reasonable to anticipate that mental health care services did catch up to meet the needs of the Swedish-born population over time, and the stable trends we observed in this population—particularly during 2021—are not the result of ongoing unmet health care needs. This argument is supported by reports showing that suicide rates – strongly linked to mental disorders – have not increased during the pandemic in the Swedish population [8]. This resilience of Swedish-born might be explained by the rapid adaptation of the health care system at the beginning of the pandemic [28], the effective social and economic support strategies during the pandemic [38], and the awareness on the mental health effect in the mitigation strategies of the Public Health agency in Sweden [39].

We found evidence that specialized mental health care use due to CMDs but not antidepressant prescription witnessed a relative slight increase during the pandemic in refugees. There have been no other studies with similar outcomes in other countries among refugees that we can compare our results with. A survey-based study from Sweden and five other European countries did not find substantial changes in symptoms of depression and anxiety during the first year of the pandemic, when comparing those who lived in a different country as their birth country compared with those who lived in their birth country [24]. However, in this study there was no pre-pandemic measurement, the study population was selected due to convenient sampling and the number of participants with immigrant background was low. Our findings suggest that it is particularly the rate of moderate to severe cases of CMDs requiring specialized care there might be a slight increase in refugees during the pandemic. However, given the known underutilization of specialized mental health care in refugees [19] and the disruptions in psychiatry service provision experienced, at least initially, the observed increase in specialized mental health care use might still mask an increase in the treatment gap in refugees during the pandemic.

Several factors might have been contributing to an increase in mental health care need in refugees during the pandemic. A recent study found that poor living conditions, pre-existing social deprivation, language and economic difficulties were associated with a higher frequency of CMDs symptoms in refugees during the pandemic [40]. It is important to disseminate and implement the already available culturally sensitive public health interventions for resettled refugees in situations of future pandemics and other public health crisis [41]. In a country like Sweden, with large immigrant populations where approximately half of the foreign-born were refugees on arrival to their new country [42], strategies to meet the mental health care needs of refugees are of outmost Public Health importance. The current cost-of-living crisis and potential long-term consequences of the pandemic make this an even more pressing topic.

We also found that the increase in specialized mental health care use due to CMDs was stronger in socially vulnerable groups of refugees such as those who have a lower attachment in the labor market or with low education. This economically vulnerable group might have been affected more seriously by the adverse consequences of the pandemic with further exposure to economic strains leading to worsening mental health [18, 22, 43, 44]. Contrary to specialized mental health care use, antidepressant prescription did not change during the pandemic in these refugees. Refugees are known to have much lower rates of initiation and persistence of treatment with antidepressants than their native counterparts and low education is one of the factors associated with particularly low treatment rates [45, 46]. The results from the sensitivity analysis showed a difference in change of antidepressant prescription between the group that had lived in Sweden five years or less and those who had lived in Sweden longer. The first group, with shorter stay in Sweden, had a larger, negative, change in trends indicating that this group might have been more affected by the pandemic in terms of accessing health care for prescription of antidepressants. Time in the new host country is important in terms of learning the language and navigating the social and health care system. Previous research in the Swedish context has showed that longer duration of residency is associated with use of outpatient psychiatric care and risk of CMDs [47, 48]. Reasons for these rates include lack of knowledge about mental disorders and their treatment, negative attitudes regarding treatment with antidepressants and possible side effects. These attitudes might not have changed over the relatively short period of the pandemic and socially vulnerable groups of refugees might have still been reluctant to accept antidepressant treatment. This, in combination with pandemic-related delays in seeking health care, might have led to the development of a more severe symptomatology finally necessitating specialized instead of primary health care. Tailor-made health literacy programs are warranted for this particularly vulnerable group.

### Strength and limitations

The major strength of this study is the use of nationwide register data covering all registered individuals in Sweden before and during the pandemic, that minimizes selection bias [49]. Moreover, the good quality of the registers ensures high diagnostic validity [50] and practically no loss to follow-up. Some limitations also must be mentioned. First, we defined the interruption of the time series as the outbreak of the pandemic for the first quarter of 2020. Within this period several important events that might generate stress and anxiety in the population had happened, and different mitigation strategies had been announced [51]. Therefore, the interruption is rather thought of as a complex event with several components. In this study we could not disentangle the contributions of the specific components on mental health outcomes. Second, information on antidepressant prescription should be interpreted with caution as a proxy of CMDs as we had no information on indication of the medication. Third, refugees are a diverse group regarding cultural, health and socioeconomic factors and regarding migration policy-related issues, such as granted permanent vs temporary residence permit [52, 53]. It is possible that there are sub-groups of refugees who might have been affected more during the pandemic with regard to their risk of a subsequently higher need of mental health care. Such sub-group analyses could not be performed in this study. Furthermore, undocumented refugees, who form a particularly vulnerable group with little access to health care, were not covered by this study. Therefore, our results cannot be generalized to this group. Finally, our findings might also have limited generalizability to other age groups and countries with different social and health insurance systems as well as different public health, social and economic mitigation strategies during the pandemic.

## Conclusions

In this population-wide register-based study, we found that specialized mental health care use due to CMDs, compared to before the pandemic, slightly increased among refugees, with a more pronounced increase among socially deprived groups, such as those with a lower labor market attachment and/or had low education. There were no changes observed among Swedish-born and in prescribed antidepressant medication in any of the observed groups. Overall, our results support the hypothesis of a mental health resilience of the general Swedish-born population during the pandemic in Sweden, but also highlight the importance of monitoring the mental health of vulnerable populations, identify those with increased need of mental health services and focus public health efforts to these groups.

## Supplementary Information

Supplementary Material 1. Description of the Swedish social insurance system. Table 1. Incidence rates (IR) of specialised psychiatric health care use per 1000 py in each quarter. Table 2. Incidence rates (IR) of prescription of antidepressants per 1000 py in each quarter. Table 3. Quarterly changes of incidence rates and incidence rate ratios for outcomes observed stratified by by length of stay in Sweden. Fig. 1. Incidence rates and trends of outcomes studied among refugees, stratified by length of stay.

## Data Availability

The data used in this study cannot be made publicly available due to privacy regulations. According to the General Data Protection Regulation, the Swedish law SFS 2018:218, the Swedish Data Protection Act, the Swedish Ethical Review Act, and the Public Access to Information and Secrecy Act, these types of sensitive data can only be made available for specific purposes, including research, that meets the criteria for access to this sort of sensitive and confidential data as determined by a legal review. Readers may contact Professor Kristina Alexanderson (kristina.alexanderson@ki.se) regarding the data.
